# What the systematic review of HPV vaccine clinical study reports does, and does not, reveal: commentary on Jørgensen et al.

**DOI:** 10.1186/s13643-020-01299-5

**Published:** 2020-02-28

**Authors:** Hilda Bastian

**Affiliations:** Bond University, Robina, Australia

Getting hold of, and analysing, clinical study reports (CSRs) for a substantial number of trials of HPV vaccines was a massive undertaking. Nearly 60,000 pages worth of CSRs were amassed, with potentially unique unpublished data points in the tens of thousands. In the end, though, I believe the systematic review by Jørgensen, Gøtzsche, and Jefferson [1] demonstrates the risks of relying too much on CSRs.

Journal articles are the usual clinical trial reports that systematic reviewers gather and analyse. Articles typically condense a possibly vast amount of data and methodological detail into just a few pages, perhaps with supplementary data files. That is a highly selective process—often misleadingly so. CSRs, on the other hand, do not have space constraints. The longest CSR package included in this systematic review had 11,456 pages, and even the shortest was 357 pages long.

However, CSRs are not synonymous with comprehensive data. All trials do not have CSRs, for starters. In this review, sometimes, there was one, but the researchers could not get access to a full copy, or the terms of use were too restrictive. Even when a CSR was available, it too included some condensed data, selective results reporting, and gaps in important detail. Sometimes, when data were in the supplied CSR, there were redactions. In the end, the authors report they did not get a single complete, unredacted CSR [2].

More than 3 years after they began requesting CSRs, Jørgensen and colleagues [3] were still missing trials for over 21% of all the people who participated in eligible studies. That means there is far more than 21% missing data for some outcomes not fully reported in the available CSRs. That is such a substantial amount, that, as the authors acknowledge, getting hold of it could up-end outcomes at the margins of statistical significance.

When the authors compared meta-analyses drawn from CSR data with those from data drawn from journal articles and/or a clinical trial registry, they found no important differences [2]. Although they argue that theirs is the first study to undertake this type of comparison, there have been similar studies. Results of those I found were mixed [4–7]. From the little evidence in these, plus the new Jørgensen paper, the absence of CSR data cannot be assumed to render the estimates calculated in a systematic review based on articles unreliable.

The principal value of CSRs seems to lie in data they contain that is not available in any other document. Unfortunately, Jørgensen and colleagues’ methods paper does not include reliable estimates of the extent and importance of data missing from systematic reviews of journal publications, as the authors only compared CSR reports to a single journal article per trial [2]. That means potentially substantial amounts of published data were left out of this comparison. Similarly, the comparison of CSR reports to trial registry information is limited to a single registry, potentially overlooking additional information available on manufacturer websites and other registries.

For example, the 2018 Cochrane review of HPV vaccines included up to seven published articles for a single trial, with additional data from published pooled analyses that included the trial as well [8]. When Jørgensen et al. claim, for example, “If our systematic review of clinical study reports had relied on trial register entries or journal publications, it would have had no data for a quarter of our prespecified outcomes” [2], their findings do not relate to doing a systematic review based on all journal publications and register entries of a trial.

Turning to the outcomes of the HPV vaccines, Jørgensen and colleagues’ results show broadly similar benefits to those Arbyn et al. found from reviewing trial publications in their recent Cochrane review [8]. Take, for example, the rate of cervical lesions graded CIN 2 or higher (CIN 2+). That is the level of possible cervical cancer precursor that would typically lead to treatment for women in economically advantaged countries. CIN 2+ is used as a measure because it generally takes many years for cervical cancer to develop after HPV infection. That means these trials were not long enough to assess cancer outcomes meaningfully. For perspective, by 1996, CIN 2+ progressed to invasive cancer for about 15% of women in a UK cervical screening programme from 1976 [9]. About a third of women with cervical cancer die within 5 years in the USA [10].

The Jørgensen review found a risk ratio or relative risk (RR) of 0.81 [0.68–0.97] for CIN 2+ based on 1 to 4 years of follow-up, or about a 20% reduction (from approximately 4.9% to 3.8%) (Additional file 4, analysis 3.7). The Arbyn review found an RR of 0.79 [0.65–0.97] for women who had received at least one dose of vaccine, based on follow-up mostly around 4 years, with one trial over 8 years (analysis 3.7). That was a reduction from approximately 5.1% to 4.0% in a few years.

The most influential conclusions of this systematic review, however, are likely to be the claims about serious and rare neurological harms, based on what the authors make clear are post hoc exploratory analyses. That is extremely worrying because I believe the authors are on very shaky ground here. The conclusions are shaky not just because of the risk of missing data overturning the findings as the authors discuss. To understand how troublesome I find their claims, we need to go back into the early stages of their study.

The original protocol for the systematic review envisaged gaining access to a rich source of detailed data on adverse events from CSRs. In particular, they wanted to be able to assess the risk of two rare neurological conditions, postural orthostatic tachycardia syndrome (POTS), and complex regional pain syndrome (CRPS). They point out in their protocol that both these conditions are exclusionary diagnoses: they could only apply if other, far more likely, causes of symptoms are ruled out.

However, when the yield of data was disappointing, the authors made two amendments to their protocol [11], developing more indirect ways of trying to assess potential harms as they realised their original plans were not feasible. In my view, this undercuts the methodological rigour of their work.

This, for example, is how they ultimately arrived at something they call “harms judged as ‘definitely associated’ with” POTS or CRPS. They collected every unique term used for any recorded adverse event and put them into an Excel sheet. They asked a single clinician to code those she considered definitely associated with POTS or CRPS. The result, as the authors point out, included conditions “that do not align well with the diagnostic criteria of POTS or CRPS”, like constipation. Coded “definitely associated” was a very long list of symptoms including many kinds of common pain, conditions including food poisoning, and having tests including chest X-rays, blood tests, and ultrasounds. There did not have to be a cluster of them. These events are exceedingly more likely not to be associated with POTS or CRPS than they are to be a signal of a rare neurological condition.

The next methodological issue I found problematic was their conclusion, “we found that the vaccines caused serious neurological harms”. They had data classified as serious neurological events, but they did not know how many separate individuals experienced them. So if a person had a headache bad enough to interfere with their normal activity as well as dizziness that affected them as badly, or they had disturbed sleep (or all three), then that one person would be counted as two (or three) people with serious neurological harms.

The problems inherent in the underlying data were exacerbated by the use of statistical methods that, in my opinion, systematically distorted the presentation of rates for association with POTS and CRPS and serious neurological harms. The authors computed rates that they presented as risk ratios, and, derived from those, numbers needed to harm (NNH). Both of these statistics unambiguously require knowing how many individuals were affected by harms as a proportion of all individuals [12, 13]—data that the authors did not have. You cannot know the risk of being harmed if you do not know how many people were harmed.

The authors drew the line at continuing this method of calculation in cases where events were so common that the numerators eventually exceeded denominators. The results of a meta-analysis in these circumstances, they wrote, would be “nonsensical”. But the respective size of the data points is not what compromises these analyses. The problem is doing calculations with data points other than those the formulas require.

The authors argued that statistically exaggerating the rate of harms would be acceptable because adverse effects are likely to be under-ascertained. For example, any that might theoretically be caused by vaccine adjuvants would be hidden as the comparison groups were not placebos. They also point out that POTS and CRPS could be under-diagnosed. We cannot be sure, though, of the potential magnitude of any of this. What is certain is the rates being used to conclude vaccines cause serious nervous system harm and definite associations with CRPS and POTS are misnamed, and thus, misleading. As the authors report, their analyses show no statistically significant increase in any individual serious or fatal adverse event, or overall serious or fatal harms.

The authors also argue that events classified as serious neurological ones were so uncommon, duplication was unlikely. That is contestable, in my view, given the nature of those events (headaches, to a large extent). If the estimates are accurate, however, and also not a statistical fluke, the risk they calculated is very small: 0.15% versus 0.09%, an absolute difference of 0.06% (or six out of every 10,000).

Against that, we need to weigh the far more common harms caused by cervical cancer, and the surgical procedures used to diagnose it or rule it out. An estimated 2,000,000 women have abnormal Pap smear results in a year in the USA [14]. The National Cancer Institute estimates that 0.6% of women in the USA (or six out of every 1000) will be diagnosed with cervical cancer in their lifetime, and a third of them will die within 5 years. That would be two deaths for every 1000 women in the country [10]. Any substantial reduction in cervical cancer and its potential precursors will prevent anxiety and suffering on a very large scale.

This systematic review confirms that participants in these trials experienced a reduction in possible early signs of HPV-related cancers and the distressing surgical and non-surgical procedures undergone to treat abnormalities. Since the cut-off date for data inclusion in both the Jørgensen and Arbyn systematic reviews, a study following up trial participants for over 10 years has reported a statistically significant drop in cancer, too [15].

In practice, HPV vaccines are generally used at younger ages than in the trials, when the chance of already being exposed to the viruses is lower. Large-scale vaccination programmes and vaccination of boys might result in some herd immunity, and vaccines that protect against more strains of HPV than those in the trials are in use. Some estimate that the rate of cervical cancer in countries with high vaccination rates could be reduced by half or more in the next few years [8, 16, 17].

Publicity about safety concerns led to substantial drops in HPV vaccination in several countries [18–20]. So the stakes in discussing potential vaccine harms are high, both in the need to openly scrutinise the potential for harm and the need to do it responsibly. Only a very rigorous assessment could move us forward. I do not believe the Jørgensen et al. systematic review provides that.
